# Respiratory adenovirus infections in immunocompetent and immunocompromised adult patients

**DOI:** 10.1017/S0950268819002176

**Published:** 2020-01-03

**Authors:** S. Cederwall, L. I. Påhlman

**Affiliations:** 1 Division of Infectious Diseases, Skåne University Hospital Lund, Lund, Sweden; 2 Department of Clinical Sciences Lund, Division of Infection Medicine, Lund University, Lund, Sweden

**Keywords:** Adenoviruses, respiratory infections

## Abstract

Adenovirus (AdV) can cause severe respiratory infections in children and immunocompromised patients, but less is known about severe AdV pneumonia in immunocompetent adults. In this retrospective study, we compared respiratory tract infections and pneumonia caused by AdV in immunocompromised and immunocompetent adult patients regarding clinical presentation and severity of infection. The results show that AdV can cause severe infections in both immunocompetent and immunocompromised patients, and the clinical presentation and need for hospitalisation, mechanical ventilation and antiviral treatment were equal in both groups. No underlying risk factors for severe AdV infection in healthy individuals were identified.

Pneumonia is one of the leading causes of death due to infection worldwide, and 24.5% of community-acquired pneumonia are caused by viruses according to a meta-analysis [1]. Adenoviruses (AdV) are non-enveloped, double-stranded DNA viruses with over 50 serotypes that cause infections of the respiratory and gastrointestinal tract. Severe AdV infection with high mortality rates has been described in children and immunocompromised patients [2]. There is no approved antiviral treatment, but Cidofovir has been used off-label in severe infections [3]. Respiratory AdV infections in immunocompetent adults are mostly mild and self-limiting, but severe and fatal AdV pneumonia has been described [4–6]. However, the knowledge of AdV infections in healthy adult patients is limited and mainly derived from case reports and small studies. The aim of the present study was to compare respiratory AdV infections in immunocompetent and immunocompromised patients regarding clinical presentation, severity of infection and underlying risk factors.

Patients with a positive finding of AdV in respiratory samples (nasopharynx, throat swabs or bronchoalveolar lavage (BAL)) during the period 2013–2017 were identified through the laboratory data system at the Department of Clinical Microbiology, Skåne University Hospital. Patients <18 years and patients diagnosed and treated at a primary care facility were excluded.

The study was approved by the Medical Ethic Committee (Institutional Review Board) of Lund University (reference number 2017/68).

Patient data were extracted from medical records. Symptoms, vital signs and laboratory results at the time of admission were recorded. Co-morbidity was assessed using Charlson co-morbidity index (CCI) [7]. Patients were classified as immunocompromised or immunocompetent based on underlying diagnoses and medication. Immunocompromised was defined as neutrophil counts <0.5 × 10^9^/l, presence of active solid organ or haematological malignancy, significant immune deficiency (such as hypogammaglobulinaemia), human immunodeficiency virus (HIV) infection with CD4 counts <200/ml, or on-going treatment with chemotherapy, immune suppressive therapy (such as methotrexate, tacrolimus), high-dose corticosteroids (≥30 mg prednisolone daily) or immune active monoclonal antibodies (such as rituximab, infliximab). All patients were further classified as having respiratory tract infection (RTI) or pneumonia at the time for positive AdV testing. Pneumonia was defined as symptoms of airway infection (fever, cough, chest pain, shortness of breath) in combination with radiological findings (chest x-ray or CT scan) compatible with pneumonia. RTI was defined as symptoms of airway infection in the absence of radiological findings of pneumonia. The severity of infection was graded according to quick Sepsis Related Organ Failure Assessment (qSOFA) and the pneumonia severity score CRB-65 [8].

Comparisons between groups were made by the non-parametric Mann–Whitney *U* test or Pearson *χ*^2^ test using the Graph pad Prism7 software (Graphpad Inc., San Diego, CA, USA). Two-tailed *P* < 0.05 was regarded as statistically significant.

A total of 41 adult patients were positive for AdV in airway samples during the study period. Eight patients were excluded; six patients had no hospital visit registered and two patients were subjected to routine screening and lacked symptoms of infection. Of the remaining 33 study participants, 11 patients (33%) were classified as immunocompromised; two patients had on-going chemotherapy due to malignancy, two patients had undergone allogeneic and one autologous stem cell transplantation, two patients with cystic fibrosis had performed lung transplantation, three patients were on immunosuppressive therapy due to multiple myeloma, idiopathic thrombocytopenic purpura and rheumatoid arthritis, and one had inadequately treated HIV infection.

The immunocompromised and immunocompetent groups were comparable with regards to age, gender and smoking habits, although information about smoking status was missing in 17 (52%) of the medical records. The presence of airway diseases such as asthma and chronic obstructive pulmonary disease were equal in the two groups. Malignancy was significantly more common in the immunocompromised group (*P* < 0.001), but there was no significant difference in total co-morbidity according to CCI scores (*P* = 0.099) (Table 1).

**Table 1.** Clinical and laboratory data for respiratory AdV infections in immunocompetent and immunocompromised patients

	Immunocompetent *n* = 22	Immunocompromised *n* = 11	*P*-value
Basic patient characteristics			
Age (years)	44.5 (28.5–67)	53 (23–65)	0.98
Male gender; % (*n*)	64 (14)	45 (5)	0.46
Smoker; % (*n*)	18 (4)	9 (1)	0.64
Underlying conditions; % (*n*)			
COPD	14 (3)	9 (1)	>0.99
Asthma	5 (1)	0 (0)	>0.99
Sleep apnoea	9 (2)	9 (1)	>0.99
CCI	0 (0–3)	2 (1–5)	0.099
Clinical symptoms; % (*n*)			
Cough	59 (13)	64 (7)	>0.99
Sore throat	27 (6)	45 (5)	0.44
Myalgia	32 (7)	9 (1)	0.22
Fever	86 (19)	73 (8)	0.38
Headache	32 (7)	9 (1)	0.22
Conjunctivitis	14 (3)	0 (0)	0.53
Dyspnoea	50 (11)	18 (2)	0.13
Initial vital signs			
Temperature (°C)	37.9 (37.0–39.1)	37.1 (36.5–40.0)	0.97
Oxygenation (%)	95 (92–97)	95 (92–98)	0.84
Heart rate (beats/min)	94 (85–106)	86 (74–106)	0.37
Respiratory rate (breaths/min)	20 (17–30)	18 (15–21)	0.13
Systolic blood pressure (mmHg)	136 (119–160)	112 (106–123)	0.011*
Laboratory findings			
WBC (×10^9^/l)	9.3 (7.2–13.1)	6.2 (2.6–7.9)	0.010*
Platelet counts (×10^9^/l)	187 (151–282)	150 (93–309)	0.30
CRP (mg/l)	101 (31–148)	54 (10–149)	0.18
Co-infection bacteria; % (*n*)	23 (5)	36 (4)	0.44
Co-infection virus; % (*n*)	9 (2)	36 (4)	0.15
Severity of infection			
Pneumonia; % (*n*)	64 (14)	64 (7)	>0.99
Hospitalised; % (*n*)	64 (14)	73 (8)	0.71
Days of hospitalisation	5 (0-8)	4 (0–13)	0.58
CRB-65^a^	1 (0–2)	0 (0–0.75)	0.18
qSOFA	1 (0–1)	0 (0–1)	0.36
Oxygen treatment; % (*n*)	32 (7)	20 (2)	0.68
NIV; % (*n*)	5 (1)	9 (1)	>0.99
Intubation; % (*n*)	5 (1)	9 (1)	>0.99
Antibiotic treatment; % (*n*)	77 (17)	64 (7)	0.44
Antiviral treatment; % (*n*)	5 (1)	18 (2)	0.25

COPD, chronic obstructive pulmonary disease; CCI, Charlson co-morbidity index; WBC, white blood cell counts; CRP, C-reactive protein; CRB-65, pneumonia severity score; qSOFA, quick Sepsis Related Organ Failure Assessment; NIV, non-invasive ventilation.

Data are presented as median and interquartile range unless otherwise stated.

a Evaluated for pneumonia patients only, *n* = 21.

*p < 0.05.

The distribution of RTI and pneumonia was equal in the two groups (*P* > 0.999), and clinical symptoms were similar between immunocompromised patients and controls. Fever was the most common symptom, followed by cough and dyspnoea (Table 1). The median white blood cell count (WBC) at admission was significantly lower in the immunocompromised group with a median of 6.2 × 10^9^/l compared to 9.3 × 10^9^/l in the immunocompetent group (*P* = 0.010). Neither platelet counts nor CRP levels were significantly different (*P* = 0.301 and 0.181, respectively). Systolic blood pressure at admission was significantly lower in the immunocompromised group (*P* = 0.011), whereas all other vital signs were comparable (Table 1).

The severity of infection was equal in the immunocompetent and immunocompromised groups according to qSOFA and CRB-65 scores (*P* = 0.36 and 0.18, respectively). There were no differences between the groups in terms of oxygen treatment (32% *vs.* 20%, *P* = 0.681) or need for hospitalisation (64% *vs.* 73%, *P* = 0.709). Three patients, one immunocompromised and two immunocompetent, required non-invasive ventilation (NIV) and/or intubation. Three patients were given antiviral treatment with Cidofovir. Two of these were immunocompromised, one of whom was intubated and one managed without lung support. The immunocompetent patient that received Cidofovir needed NIV treatment. One patient in the immunocompromised group died.

Antibiotic treatment was common; 77% of the immunocompetent and 65% of immunocompromised patients (*P* = 0.438). Co-infection with bacteria was detected in 27% of all study participants, and other viruses in 18% of the patients. The distribution was equal between the groups. Two patients with pneumonia had other microbial findings that were regarded as significant; one immunocompetent patient had concomitant findings of *Mycoplasma pneumoniae* in a throat swab, and one immunocompromised patient had growth of *Streptococcus pneumoniae* and *Pneumocystis jirovecii* in BAL.

Out of the four patients that needed NIV, intubation and/or Cidofovir treatment, two had growth of *candida* in sputum, two had elevated copies of cytomegalovirus in plasma, one had findings of *Stenotrophomonas* in the bronchial brush culture, and one was positive for BK-virus in plasma.

Severe AdV infections have mostly been recognised in children and immunocompromised individuals [2, 9]. However, in this retrospective study on respiratory AdV infections in adults, only one-third of the patients were classified as immunocompromised. There were no significant differences between immunocompromised and immunocompetent patients in the clinical presentation or severity of infection, and no apparent risk factors for severe AdV infections in healthy individuals could be identified. Co-morbidity, assessed as CCI scores, tended to be higher in the immunocompromised group but did not reach statistical significance. This result was surprising, as the immunocompromised group by definition has underlying conditions that the immunocompetent group lacks, and it suggests that the immunocompetent group may have more co-morbidities other than immune suppression. However, no underlying conditions were over-represented in the immunocompetent group, and the lack of statistical significance may be explained by the low statistical power. Moreover, some of the conditions affecting immune status were not part of the CCI scoring system. As a result, some immunocompromised patients received low or no CCI scores despite severe immune disorders such as hypogammaglobulinaemia. Consequently, CCI may not represent a true assessment of co-morbidity for this group of patients.

WBC and systolic blood pressure were the only parameters that differed significantly between the groups. WBC was significantly lower in the immunocompromised group, but this is probably explained by underlying conditions rather than of the AdV infection itself. For example, patients with neutropenia due to haematological malignancy or chemotherapy were part of this group.

Co-infection with bacteria was present in 27% of the patients, which is similar to the numbers reported in other studies [10]. In two cases, the concomitant bacterial findings were regarded as significant and likely to contribute to the patients' symptoms. However, assessment of causative agent is difficult and this study does not allow interpretation of the true impact of AdV infection on clinical symptoms. Even so, co-infections were equally distributed between the two groups and do not change the conclusion that also healthy individuals can suffer from severe AdV infection.

Our study has several limitations. The number of cases is small, which may partly be explained by the low incidence of AdV pneumonia in adults [11]. However, we probably miss a large number of patients with mild AdV infection that were not tested. Testing for AdV is not part of the standard work up for pneumonia, and there was no systematic sampling of patients due to the retrospective study design. Moreover, there is a possible bias that immunocompromised patients are subjected to AdV testing more often than immunocompetent individuals, and that only the most severely ill immunocompetent patients are tested. Another limitation is that no AdV typing was performed at the time of sampling, and samples were not available for retrospective analyses. Other studies have shown that AdV-55 is common in severe infections in healthy patients [4, 6, 10, 12]. A prospective study would be needed to estimate the true incidence of AdV pneumonia in healthy and immunocompromised adults, and to establish if specific serotypes are over-represented in immunocompetent individuals.

In conclusion, this study shows that both immunocompromised and otherwise healthy patients are at risk for severe AdV infections that require antiviral and intensive care treatment. Testing for AdV and other respiratory viruses should be considered in patients with severe pneumonia where no other causative agent has been identified.
